# Using neuronal models to capture burst-and-glide motion and leadership in fish

**DOI:** 10.1098/rsif.2023.0212

**Published:** 2023-07-19

**Authors:** Linnéa Gyllingberg, Alex Szorkovszky, David J. T. Sumpter

**Affiliations:** ^1^ Department of Mathematics, Uppsala University, Uppsala, Sweden; ^2^ Department of Information Technology, Uppsala University, Uppsala, Sweden; ^3^ RITMO Centre for Interdisciplinary Studies in Rhythm, Time and Motion, University of Oslo, Oslo, Norway

**Keywords:** collective behaviour, swimming dynamics, neuronal dynamics, dynamical systems, fish behaviour

## Abstract

While mathematical models, in particular self-propelled particle models, capture many properties of large fish schools, they do not always capture the interactions of smaller shoals. Nor do these models tend to account for the use of intermittent locomotion, often referred to as burst-and-glide, by many species. In this paper, we propose a model of social burst-and-glide motion by combining a well-studied model of neuronal dynamics, the FitzHugh–Nagumo model, with a model of fish motion. We first show that our model can capture the motion of a single fish swimming down a channel. Extending to a two-fish model, where visual stimulus of a neighbour affects the internal burst or glide state of the fish, we observe a rich set of dynamics found in many species. These include: leader–follower behaviour; periodic changes in leadership; apparently random (i.e. chaotic) leadership change; and tit-for-tat turn taking. Moreover, unlike previous studies where a randomness is required for leadership switching to occur, we show that this can instead be the result of deterministic interactions. We give several empirically testable predictions for how bursting fish interact and discuss our results in light of recently established correlations between fish locomotion and brain activity.

## 1. Introduction

A wide range of mathematical and computational models have been proposed to capture the collective motion of fish schools [1–7]. These models are often referred to collectively as self-propelled particle models (SPPs), with the name coming from a seminal paper by Vicsek *et al.* [8]. The Vicsek model assumes that each fish (or particle) moves with constant speed, while its direction is updated at each time step to be closer to the average direction of individuals within its neighbourhood. A noise term is added to model uncertainty or error in the fish’s direction. Variations of this canonical model extend it to include repulsion, attraction and other social interactions [9–12].

These models reproduce many of the observed properties of large fish schools [12,13], but do not always capture the interactions of smaller shoals. One difference is that the assumption that the fish moves with constant speed is not typical of the swimming behaviour for many species of fish. For example, zebrafish [14], koi carps [15], guppies [16], cod [17], rummy-nose tetra fish [18] and many other species swim by alternating between accelerated motion and powerless gliding [19]. Even other animals, such as spiders, beetles and lizards have this type of intermittent locomotion [20]. For swimming animals, intermittent motion is often referred to as burst-and-coast or burst-and-glide. We use the latter term in what follows. Models of burst-and-glide behaviour have been actively developed in the biomechanics community for decades [17,19,21–26]. It has been shown that there are energetic advantages of such movements, when compared with constant swimming speed. The energetic cost of swimming is minimized during the glide phase, where the body is rigid and the fish decelerate due to water resistance [19]. Once a low velocity is reached, the burst phase starts again and the fish accelerates to a maximum velocity. The cycle is then completed and the fish glides again. Burst-and-glide movement is pivotal to detect and quantify social interactions between individual fish [16]. Herbert-Read *et al.* observed that, when swimming in pairs, guppies respond to the other fish by accelerating their motion when the other fish is nearby. Pairs of fish do not update their speed continuously, but at discrete time points, making Vicsek type of models unsuitable for understanding the social dynamics of small shoals of guppies. Fish living in high predation areas have more pronounced acceleration and deceleration responses [16]. Moreover, Kotrschal *et al.* found that the high burst speed in response to neighbours evolves when subjected to artificial selection [27].

Another aspect which is not included in the most common self-propelled particle models, but is found in fish schools of several species, is leadership. Schaerf *et al.* showed that for pairs of freely exploring eastern mosquitofish, it is possible to categorize the fish into leaders and followers—some fish takes the role of leaders and will on average spend more time in front than behind the other fish [28]. In this case, one particular fish is the leader. However, other studies show that fish change leader–follower roles. For example, Nakayama *et al.* show that leadership in pairs of stickleback changes over time [29], and Milinksi shows tit-for-tat like strategies in pairs of swimming sticklebacks when confronting a potential predator [30].

As we outline above, even though studies of interacting burst-and-glide behaviour are becoming more common, most studies of the burst-and-glide responses are of isolated fish, with a focus on understanding the energetics. Even though some self-propelled particle models assume variable speed on the part of the fish [31], they do not necessarily model burst-and-glide behaviour. Calovi *et al.* made an interesting start in this direction by incorporating burst-and-glide behaviour into an SPP model [32]. They have developed a model of two-fish dynamics where the speed of individual fish is updated at discrete-time points and the heading angle is updated due to attraction and alignment with the other fish. However, this model does not capture the observations from Herbert-Read *et al.* and Kotrschal *et al.* that there is a direct response in speed, and not just heading angle, when two fish interact with each other [16,27]. Thus, developing a simple model for speed response of fish interactions remains an open question.

In order to incorporate social information into burst-and-glide dynamics, it is necessary to consider how burst timings and amplitudes are governed in the brain. Continuous rhythmic patterns of movement in fish and other vertebrates are achieved by a neural circuit known as a central pattern generator, with afferent feedback as an important modulating component [33]. Recent work indicates that the control of episodic bursting in larval zebrafish is similarly distributed, yet separable from the system that coordinates the bursts themselves [34]. Although the exact mechanism for this control is still unclear, there is ample evidence for an internal burst/glide state that is subject to modulation by sensory input.

There is also an intriguing similarity between the burst-and-glide of fish and the burst-and-recover phases of neurons. The FitzHugh–Nagumo model [35,36], a well-known model of neuronal firing, is a simplification of Hodgkin and Huxley’s Nobel prize winning model for action potentials in neurons [37]. The FitzHugh–Nagumo model has two variables, *V* and *W*, which do not explicitly relate to specific chemical and electrical properties of neurons. Instead, *V* models the membrane potential and *W* is thought of as a recovery variable. The neuron dynamics in the FitzHugh–Nagumo model is given by the following equations:

1.1 $$\frac{dV}{dt}=V-\frac{V^{3}}{3}-W+c$$

and

1.2 $$\tau \frac{dW}{dt}=V+a-bW.$$

The cubic-shaped dependence in the first equation captures the essential bistability of the membrane potential *V* and, for certain parameter values, the system displays intermittent switching between firing and recovery, at a rate governed partially by the slow exponential decay of the recovery variable *W*. The constant *c* represents a tonic (i.e. constant) input to the neuron.

An interesting property of the FitzHugh–Nagumo model, is that when two FitzHugh–Nagumo model neurons are coupled, the system displays chaotic dynamics [38]. The evidence for chaos in neural mechanisms is extensive [39–41]. Chaotic dynamics occur on both macroscopic and microscopic scales in brain dynamics [42] and in both humans and animals [43,44]. Neuroscientific studies show that chaos is also essential for many neural mechanisms. For example, Ohgi *et al.* show that chaotic dynamics are fundamental in the learning and control of the dynamical interactions between brain, body and environment in human infants [45].

There are currently no existing self-propelled particle models with internal neural states explicitly defined, despite sensory information being increasingly a focus for collective motion studies [46–49]. Instead, models tend to be based on heuristic ‘rules of interaction’ where the ability to turn sensory information into actions, such as averaging neighbour headings to obtain a turning angle, is assumed to be instantaneous and left out of the model. More complete biological models, on the other hand, are difficult to scale up to multiple agents, and the high complexity makes it difficult to connect specific mechanisms to collective outcomes. However, there are intriguing starts in this direction made by O’Keeffe *et al.*, where an SPP model is combined with the Kuramoto model [50], and Wang *et al.* have extended the model of Calovi *et al.* [32] to multiple fish [51].

Less direct analogies have previously been made between collective motion and activities in the brain. For example, Marshall *et al.* draw parallels between decision-making in ants and decision-making in primate brains [52], Passino *et al.* show that some main characteristics of cognition in brains of vertebrates are also present in swarms of honey bees [53], and on a more general level, Ribeiro *et al.* discuss the connections between animal group dynamics and networks in brains [54]. There are also existing models connecting sensory systems to neuronal dynamics. For example, Kuniyoshi *et al.* have presented a mathematical model coupling the sensory system in muscle spindles to neuronal dynamics in human infants [55]. Furthermore, there are indications of correlations between different swimming activities and activities in fish brains [56,57], although as yet there is no proven link between burst-and-glide and neuronal bursting.

In this article, we consider the possibility that even simple deterministic neural models are rich enough in dynamics to capture seemingly stochastic burst-and-glide motion patterns in fish, and that this can be used as a basis for self-propelled particle modelling. We begin with a two-variable dynamical model, inspired by the FitzHugh–Nagumo model, one representing the internal state and the other representing speed, as a model for a single fish. We then introduce social interaction, and characterize the behaviour of a pair of fish as a function of key parameters. The aim of our study is primarily to investigate the mechanisms at work [58–62], rather than to compare the model in detail with data. Nonetheless, we show that the patterns of interactions created in the model mimic those observed in single and pairs of guppies. We discuss several testable hypotheses arising from our model.

## 2. Single-fish model

We modify the Fitzhugh–Nagumo model so that recovery is governed by sensory feedback of the fish speed rather than internal dynamics. Our model has two variables: the speed of the fish, *v*, and the burst potential, *b*, which determines whether the fish is bursting or gliding. Fish are known to adjust their bursting times to conspecifics [63] and our variable *b* gives an abstract representation of the fish’s responsive state. We model motion along a line, so that *v*(*t*) defines the motion entirely. The velocity itself is subject to propulsive force and drag

2.1 $$\frac{dv}{dt}=g(b)-kv.$$

Here *g*(*b*) ≥ 0 is the propulsive force, which is a function of the internal state. To approximate the bursting impulse, we assume this function increases in a step-like manner from zero towards a positive constant at a critical value of *b*. We begin with linear drag with coefficient *k*, which is a suitable approximation for fish with low Reynolds number (≲1000) such as Trinidadian guppies or zebrafish (a quadratic drag is often included in models of larger fish [17,21,23–25] and we will consider this in §3.5). The fish therefore alternates between gliding (i.e. decelerating) when d*v*/d*t* = *g*(*b*) − *kv* < 0, i.e. when *g*(*b*) < *kv*, and bursting (i.e. accelerating) when d*v*/d*t* = *g*(*b*) − *kv* > 0, i.e. when *g*(*b*) > *kv*. The full model is now

2.2 $$\frac{db}{dt}=b-\frac{b^{3}}{3}+c-v$$

and

2.3 $$\frac{dv}{dt}=g(b)-kv,$$

where for *g*(*b*) we choose the sigmoidal inverse tangent function

2.4 $$g(b)=a\left(\frac{\arctan(z_{1}(b-b_{0}))}{\pi }+\frac{1}{2}\right).$$

This model again resembles the FitzHugh–Nagumo model, but with a sigmoidal rather than linear function for *g*(*b*). In fact, although the functional forms are relatively simple, the shapes of these functions (cubic and sigmoidal, respectively, in the fast variable, and linear in the slow variable) are very common in fast–slow type neural models [64].

The equation for the internal state *b* has one main parameter, *c*, which controls the bursting rate and amplitude. This parameter represents the tonic control of frequency and amplitude of motion that is known to come from the brain stem [33,65].

**Figure 1.**
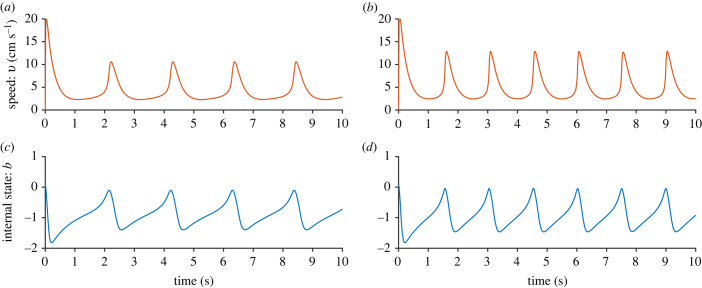
One-fish model with *k* = 0.4, *a* = 15 cm s^−2^, *z*_1_ = 50 and *b*_0_ = 0. Panels (*a*) and (*b*) show the time series of the speed and (*c*) and (*d*) show the time series of the internal state. In (*a*) and (*c*), *c* = 0.95, and in (*b*) and (*d*), *c* = 1. We see that a higher value of *c* results in a higher frequency and a higher amplitude of the speed.

**Figure 2.**
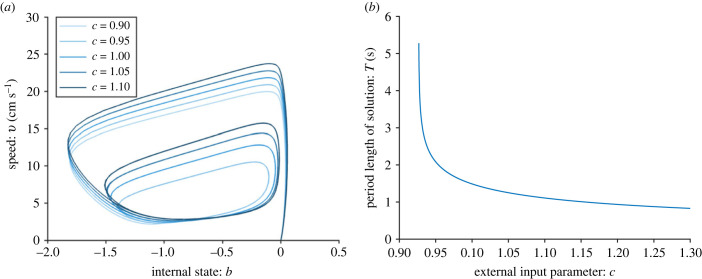
One-fish model with *k* = 0.4, *a* = 15 cm s^−2^, *z*_1_ = 50 and *b*_0_ = 0. Panel (*a*) shows the phase plane for five different values of *c*, as shown in the legend. For *c* = 0.9, the dynamical system has a stable equilibrium, resulting in constant speed. For *c* = 0.95 and larger, there is a stable limit cycle. As *c* increases, the amplitude of the speed increases. Panel (*b*) shows the period length as a function of *c*. Increasing *c* leads to decreasing period length and thus increasing frequency.

In figure 1, we see the time series for the one-fish model for two different values of *c*. Both *c* = 0.95 and *c* = 1 result in burst-and-glide swimming, but *c* = 1 gives a higher frequency and amplitude of the speed. In the modelled scenario of a fish swimming down a channel, the fish accelerates approximately 4 cm down and then glides approximately 4.3 cm (for *c* = 0.95). For *c* = 1, the fish accelerates approximately 2.3 cm, and then glides approximately 4.8 cm. Figure 2*a* shows phase plane trajectories for five different values of *c*. For *c* = 0.9, there is a stable equilibrium at (b*, v*) ≈ ( −1, 2.3), meaning that neither of the fish move with intermittent burst-and-glide locomotion, but with a constant speed of approximately 2.3 cm s^−1^. As *c* is increased to *c* = 0.95, there is a limit cycle, meaning there is burst-and-glide behaviour. As *c* increases further, the limit cycle increases, leading to increasing amplitude of the speed. In figure 2*b* we see period length of the limit cycle as a function of *c*. We see that there is a Hopf bifurcation at *c* ≈ 0.927, below which there is a stable equilibrium. On the other side of the bifurcation, the limit cycle period *T* rapidly decreases from infinity before approaching a value of the order of 1 s for *c* > 1. This means that the higher the value of *c*, the more often the fish bursts.

## 3. Two-fish model

### 3.1. Coupling between fish

With our single-fish model as a starting point, we now build a two-fish model. As for the single-fish model, the two fish are assumed to move along a one-dimensional line, e.g. swimming up a channel, and are at the points *x*_1_(*t*) and *x*_2_(*t*), respectively, at time *t*. We let *v*_1_(*t*) and *v*_2_(*t*) be the velocity of the two fish, at time *t*, and *b*_1_(*t*) and *b*_2_(*t*) be the internal state of the fish as described in §2.

As position, not velocity, has shown to be most important in determining the reactions of fish [16,63], we couple the equations for the two fish using a response function *f*(*x*), where *x* is the distance (negative when the other fish is behind, positive when in front) between the fish. As we saw in the single-fish model, the burst frequency and the maximum speed increases as *c* increases. As we want the fish to burst when it is behind another fish, the fish are coupled in the way that their internal state is affected by the distance between the fish, making *f* act as an extra boost on *c*

3.1 $$\frac{db_{1}}{dt}=b_{1}-\frac{b_{1}^{3}}{3}+c+f\;(x_{2}-x_{1})-v_{1},$$

3.2 $$\frac{db_{2}}{dt}=b_{2}-\frac{b_{2}^{3}}{3}+c+f\;(x_{1}-x_{2})-v_{2},$$

3.3 $$\frac{dv_{1}}{dt}=g(b_{1})-kv_{1},$$

3.4 $$\frac{dv_{2}}{dt}=g(b_{2})-kv_{2},$$

3.5 $$\frac{dx_{1}}{dt}=v_{1}$$

3.6 $$\mathrm{and}\;\;\;\;\;\frac{dx_{2}}{dt}=v_{2}.$$

We want the follower fish to try to catch up with the leader. Thus, for positive *x*, *f*(*x*) is relatively large. Since most fish have wide peripheral vision, the fish will also react if the other fish is just behind, meaning that *f*(*x*) is also positive (but smaller) for *x* less than zero. It approaches zero for negative values of *x*. To model this, we use the following sigmoid function:

3.7 $$f\;(x_{2}-x_{1})=d\frac{1}{1+\exp(-z_{2}(x_{2}-x_{1}-x_{0}))}.$$

This is a logistic function which has $f\;(x)=\frac{d}{2}$ at the threshold *x*_0_ and approaches *d* as *x* becomes large, and 0 as *x* goes to −∞. The shape of *f*(*x*_1_ − *x*_2_) is illustrated in figure 4*a*. The parameter *z*_2_ determines how steep the threshold response is: a high value of *z*_2_ gives a steeper, more sudden, threshold response, whereas a low value gives a shallower threshold. Since most fish have a wide peripheral vision in our model, we want a relatively less steep threshold response—a fast switch would mean that *f*(*x*) ≈ 0 for *x* < 0, and thus entail no peripheral vision. In our simulations, we set *x*_0_ = 15 cm and *z*_2_ = 0.15 cm^−1^. This means the fish have responsiveness up to approximately 5 cm behind, but will react more strongly when the other fish is swimming in front. From the single-fish model, we know that the burst-and-glide dynamics are highly sensitive to small fluctuations of the value of *c*. Thus *d* takes values of size approximately 0.01. Since *f*(*x*) → *d* as *x* → ∞, the sigmoid function assumes that the fish would interact with the other fish infinitely far in front. This is, of course, an unrealistic assumption over large distances—a fish would not interact with a fish 50 m in front—but since we are modelling short-range interactions, this assumption can be used as a simplification for now.

### 3.2. Temporal dynamics

**Figure 3.**
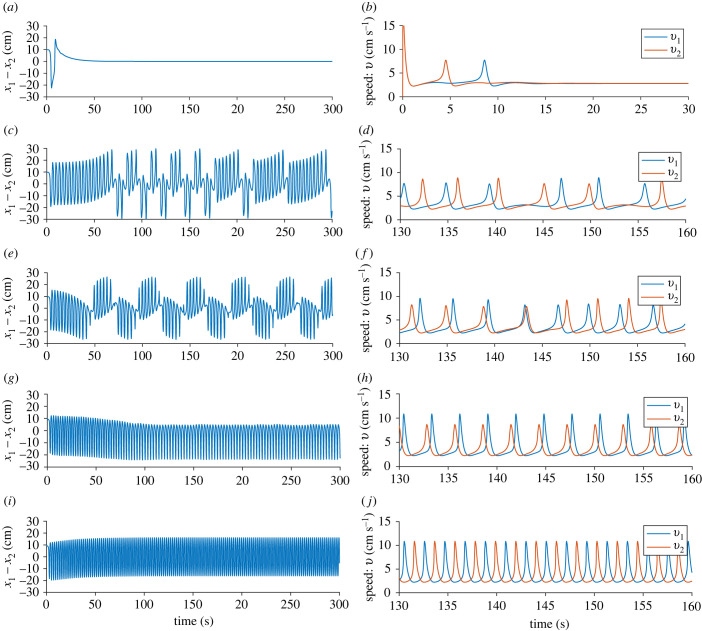
Two-fish model with *a* = 15, *c* = 0.9255, *z*_2_ = 0.15, *x*_0_ = 15, *b*_0_ = 0, *z*_1_ = 50 and *k* = 0.4. In (*a*) and (*b*) where *d* = 0.005, after one speed burst each, both fish move with constant speed and stay exactly parallel; in (*c*) and (*d*) where *d* = 0.01, there is aperiodic leadership switching; in (*e*) and (*f*) where *d* = 0.02, there is periodic leadership switching; in (*g*) and (*h*) where *d* = 0.05, there is always one fish that is the leader; and in (*i*) and (*j*) where *d* = 0.07, there is no leader.

We now examine the temporal dynamics of the two-fish model. We found five different model behaviours when we varied *d*: (i) constant swimming speed with no distance between the fish, (ii) aperiodic leadership switching, (iii) periodic leadership switching, (iv) one leader–one follower swimming, and (v) no leader swimming. These are shown in figure 3, where we see time series of the difference in distance (*x*_1_ − *x*_2_) and the speed of each fish (*v*_1_ and *v*_2_) for five different values of *d*. In figure 3*a*,*b*, where *d* = 0.005, we see that both fish undergo one burst each, and then reach an equilibrium speed of approximately 2.8 cm s^−1^. At this equilibrium, the distance between the fish quickly also reaches an equilibrium at *x*_1_ − *x*_2_ = 0. This corresponds to a steady, non-bursting swimming on the part of the fish.

When *d* is increased to 0.01 (figure 3*c*,*d*) both fish exhibit burst-and-glide motion. The fish undergo aperiodic leadership switching: the leadership of the fish alternates without any regular periodicity. The speed of each fish (figure 3*d*) is displayed from *t* = 130 to *t* = 160. In the beginning of this time series, fish 1 is the leader. Fish 1 bursts approximately 1 s before fish 2, and fish 2 has a higher maximum speed in order to try to catch up. At *t* ≈ 143 s there is switch in leadership and fish 2 bursts 1–2 s before fish 1, while fish 1 has a higher maximum speed in order to try to catch up. After three bursts each, there is yet another leadership switching, and fish 1 becomes the leader again.

Increasing *d* even further to 0.02 (figure 3*e*,*f*), the leadership switching happens periodically: after approximately seven bursts, the fish switch leader–follower roles. There is a leadership switching at *t* ≈ 144 s, where fish 1 becomes leader instead of fish 2. In the time series of the speed of the fish (figure 3*f*), we see that before the switching, fish 1 nearly catches up with fish 2, bursting approximately 1 s after, with higher maximum speed than fish 2. The burst by fish 1 happens closer and closer to the bursts of fish 2 on each cycle of burst and glide, and at *t* ≈ 143 s, fish 1 and fish 2 burst almost simultaneously, with the same maximum speed. After this, leadership switches so that fish 2 is behind and catching up to fish 1. Now fish 2 has a higher maximum speed and burst approximately 1 s after fish 1.

For *d* = 0.05, there is always one leader: whenever the following fish starts to catch up with the leader fish, the leader burst again, which we see in figure 3*g*. In figure 3*h*, we see that the follower fish (fish 1) has a higher maximum speed than fish 2, in order to try to catch up. Even though the follower fish has a higher maximum speed, the distance swum after one burst-and-glide period is approximately the same for both the follower and the leader fish.

For *d* = 0.07, there is no leader, instead both of the fish burst and glide with the exact same maximum speed, but take turns in who is bursting and who is gliding (figure 3*i*,*j*). This results in a distance between the fish that oscillates between approximately −16 cm and approximately 16 cm, with the mean distance between the fish equal to 0.

**Figure 4.**
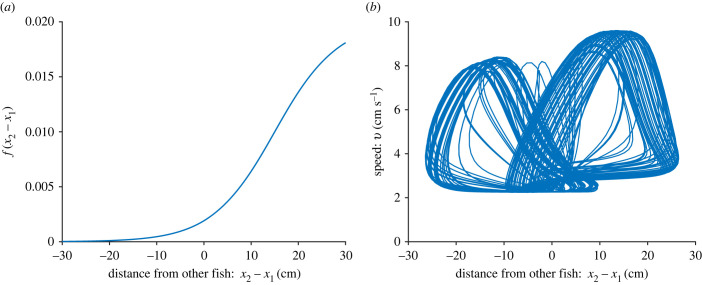
Two-fish model with *a* = 15, *c* = 0.9255, *z*_2_ = 0.15, *x*_0_ = 15, *b*_0_ = 0, *z*_1_ = 50 *k* = 0.4, and *d* = 0.02. In (*a*) the function *f*(*x*_2_ − *x*_1_) is shown, meaning that the *x*-axis shows the distance from fish 1 to fish 2 and in (*b*) we see the phase plane for the speed of fish 1 and the distance from fish 1 to fish 2. A positive distance indicates that fish 2 is in front of fish 1, and a negative distance that fish 1 is in front of fish 2.

Another way to look at these interactions is to consider the speed–distance phase plane. In figure 4*a*, we see the response function *f*(*x*_2_ − *x*_1_) as a function of *x*_2_ − *x*_1_ and in figure 4*b* the speed–distance phase plane is plotted. We notice that while the response function is asymmetrical, a maximum speed is reached both when the fish is in front of and behind the other fish. However, the maximum speed is higher when the other fish is behind than in front, which is also what we see in figure 3*f*.

### 3.3. Qualitative comparison with data

The aim of this paper is to investigate whether the model we propose qualitatively reproduces the type of motion we see between real fish. The behaviour in all of the phases are found in experiments of pairs of fish. For example, the intermittent leadership switching in our model corresponds to stickleback swapping leadership roles [29]; the constant leader–follower dynamics for higher values of the coupling parameter corresponds to the leader–follower relationship found in, for example, pairs of eastern mosquitofish [28]; and the tit-for-tat like swimming is similar to the behaviour demonstrated by Milinksi [30].

To make a further qualitative comparison between the model and pairs of fish swimming together, we compared properties of the motion of pairs of guppies (*Poecilia reticulata*) with those same properties in the model. Empirical data originated from a subset of the data used in [16], where pairs of fish were allowed to explore an open rectangular arena (1000 × 900 mm) with a depth of 45 mm for approximately 16 min. Trials were filmed with a camera recording at 1920 × 1080 pixels at 24 frames s^−1^. Fish in the videos were tracked using the software CTrax and tracking errors corrected with the associated Fixerrors GUI in Matlab, providing coordinates of the centre of mass and heading (in radians) of each fish on each frame. Trajectories were analysed when fish were more than 100 mm from the corners of the arena and when the difference in heading between the pair was less than 45${\;}^{\circ }$. Full details of the empirical methods can be found in [16].

**Figure 5.**
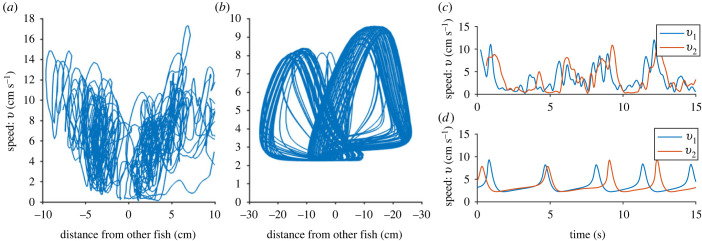
Model comparison with data of Trinidadian guppies (*Poecilia reticulata*) from [16]. (*a*) shows the phase plane of the distance between the two fish and the speed of one fish, compared with (*b*) the same phase plane for the model simulation for parameters set as *a* = 15, *c* = 0.9255, *z*_2_ = 0.15, *x*_0_ = 15, *b*_0_ = 0, *z*_1_ = 50 *k* = 0.4, and *d* = 0.02. In (*c*) (data) and (*d*) (model) we see a comparison of the speed of the two fish as a function of time for a period of 15 s. Data are from the median trial out of all female pairs in terms of activity (quantified by median speed over the trial). All time points are included where both fish are more than 100 mm away from corners of the arena, and with a difference in heading of less than 45${\;}^{\circ }$.

In figure 5, we see a phase plane plot based on data from [16], compared with the model, together with a short time series from both the model and data from the same dataset. We see that the model produces a symmetrical phase plane just as the data from the experiments. However, the minimum speed for the model is higher and the maximum speed is lower than the speed in experiments. In the short time series comparison, we see that the data from the experiments are noisier, but have approximately the same amount of bursts and glides within 15 s.

### 3.4. Role of parameters in dynamics

We now further investigate the dynamics for the different types of swimming behaviour. Since the first swimming behaviour, constant swimming speed with no distance between the fish, consists of an equilibrium point for both speed of the fish and distance between the fish, we will not investigate this swimming behaviour in our further analyses. For the other dynamics, we will use a similar technique as first described by Lorenz [66]. When studying what later became known as the Lorenz equations, he plotted the maximum value of his *z* variable on consecutive loops around the chaotic attractor. In doing so, he found that the maximum obeyed something similar to a tent map [66], which is known to produce chaotic dynamics.

**Figure 6.**
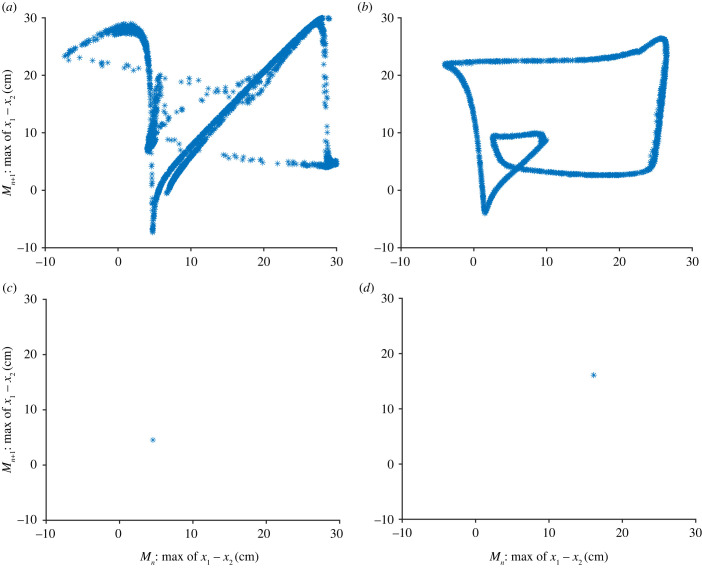
Lorenz map for two-fish model with *a* = 15, *c* = 0.9255, *z*_2_ = 0.15, *x*_0_ = 15, *b*_0_ = 0, *z*_1_ = 50 and *k* = 0.4, for maximum value. In (*a*) *d* = 0.01, in (*b*) *d* = 0.02, in (*c*) *d* = 0.05 and (*d*) *d* = 0.07.

Inspired by this approach, we find the peaks (maximum) of the difference in distance between the fish, *x*, and then plot the value of the *n*th maximum, *M*_*n*_, against the next maximum *M*_*n*+1_. These plots are shown in figure 6. For our system, this approach does not give a tent map for any of the parameter values, but it does give a clearer impression of the dynamics. In figure 6*a*, we see that the Lorenz map for *d* = 0.01 has a complicated structure most likely due to the non-periodic/chaotic dynamics. This is in contrast to figure 6*b*, when *d* = 0.02, where the Lorenz map shows a periodic pattern as the plot forms a closed loop. In figure 6*c*,*d*, i.e. when *d* = 0.05 and *d* = 0.07, respectively, the maximum values have reached an equilibrium point at 4.53 and 16.1 cm, respectively.

**Figure 7.**
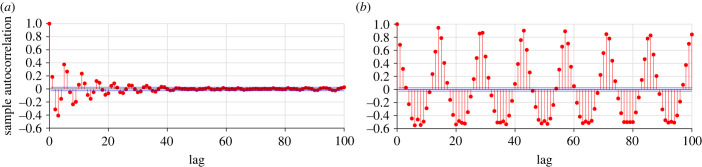
Autocorrelation for peaks of distance difference with *a* = 15, *c* = 0.9255, *z*_2_ = 0.15, *x*_0_ = 15, *b*_0_ = 0, *z*_1_ = 50 and *k* = 0.4. In (*a*) *d* = 0.01, and in (*b*) *d* = 0.02.

Another approach for investigating the differences between the various behaviours is to look at the autocorrelation function (ACF) for the peaks of the distance difference, *M*_*n*_. In figure 7, we plot the autocorrelation function of the peaks of distance, *M*_*n*_, for *d* = 0.01 and *d* = 0.02. When *d* = 0.01 (figure 7*a*), there is a weak periodic pattern of length 2–3 in the ACF. This is most likely due to the fact that many leadership switchings happen after two–three bursts, but apart from that, there is no clear periodic structure. In figure 7*b*, where *d* = 0.02, we see a clearly periodic pattern in the ACF. The periodicity is of length approximately 14, which corresponds to leadership switching after approximately seven bursts. Since the peaks of the distance difference for both *d* = 0.05 and *d* = 0.07 consist of one equilibrium point, we do not analyse the ACF for these values of *d*.

**Figure 8.**
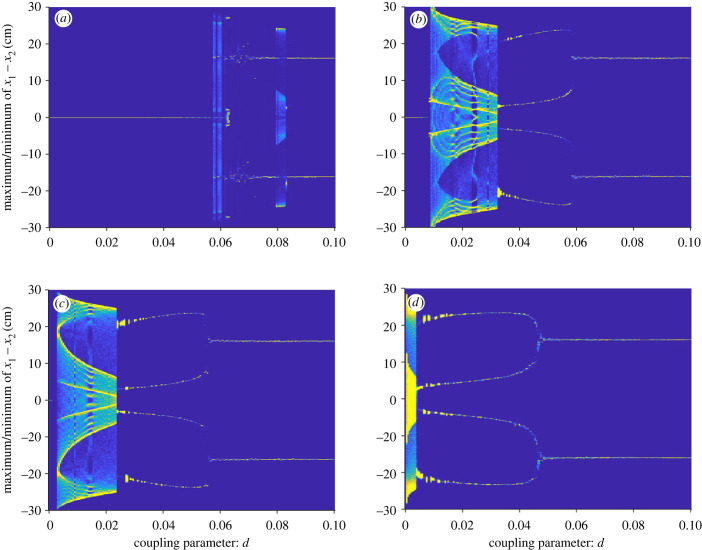
Heat plot bifurcation diagram with *d* as bifurcation parameter for two-fish model with *a* = 15, *z*_2_ = 0.15, *x*_0_ = 15, *b*_0_ = 0, *z*_1_ = 50 and *k* = 0.4. In (*a*) *c* = 0.92, (*b*) *c* = 0.9255, (*c*) *c* = 0.9265 and (*d*) *c* = 0.95. The bifurcation parameter *d* takes values between 0 and 0.1, with step size 0.0005. For each value of *d*, we simulate 10 times for 20 000 steps, each time with initial conditions $\left(b_{1}(0),b_{2}(0),v_{1}(0),v_{2}(0),x_{1}(0),x_{2}(0))=(0,0,0,0,x_{1}^{\mathrm{start}},0\right)$, where $x_{1}^{\mathrm{start}}$ is a random number between −20 and 20 cm. The distance between the fish is plotted in 701 bins, meaning that each bin is of size 0.1 cm. This allows us to create a two-dimensional histogram with 701 × 201 bins.

To get further understanding of how the model is affected both by the coupling parameter *d*, and the external input parameter *c*, we also looked at ‘heat map’ bifurcation plots with *d* as a bifurcation parameter, for different values of *c* (figure 8). Each subplot is produced by, for each value of *d*, simulating the dynamical system 10 times, with random initial position for fish 1. For each iteration, find the maximum points of the difference in distance, *M*_*n*_, and the minimum points of the difference in distance, *N*_*n*_. These are stored in a histogram with values ranging from −35 to 35 cm, from which we make a heat plot of this two-dimensional histogram, where colour indicates how frequent a value is.

In figure 8*a*, we see that when *c* = 0.92, both fish move with constant speed and with no separation between them until *d* ≈ 0.058, after which the system displays aperiodic leadership switching until *d* ≈ 0.07, where there is no leader or follower, and instead the fish take turns in bursting and gliding. Increasing *c* to 0.9255 (figure 8*b*), both fish move with constant speed and with no separation in distance between them until *d* ≈ 0.0085, when they instead display aperiodic leadership switching. When *d* exceeds 0.017, the system shifts to periodic leadership switching, which is retained until *d* ≈ 0.0325. For 0.0325 < *d* < 0.058, there is always one leader and one follower, where the leading fish is dependent on the initial conditions. After that, there is no leader or follower, instead the fish have swimming dynamics as shown in figure 3*i*,*j*. Increasing *c* to 0.9265, the system goes immediately from no separation in distance to periodic leadership switching when *d* ≈ 0.0035. After that, there is one leader–one follower dynamics, where the leading fish is dependent on the initial conditions, until *d* = 0.53, after which there is no leader or follower. When *c* = 0.93, the one-fish model has passed the bifurcation point. Thus, there is always burst-and-glide swimming, no matter the value of *d*. For *d* ∈ [0, 0.005], there is periodic leadership switching and after that the system exhibits one leader–one follower dynamics until *d* = 0.0445, where there is no leader or follower.

From all these bifurcation diagrams, we see that there is a quite narrow range of values for the coupling parameter that give rise to interesting leader–follower dynamics—when *c* = 0.9255 all four behaviours are found within the interval [0.008, 0.065] of the coupling parameter *d* (figure 8*b*). The range of values for the external input parameter *c* is even narrower: when *c* is decreased by just 0.0055 from *c* = 0.9255 to *c* = 0.92, the bifurcation diagram in figure 8*a*, shows that the model does not display one leader–one follower dynamics for any values of *d*, and when *c* is increased by just 0.0045 from *c* = 0.9255 to *c* = 0.93, the bifurcation diagram in figure 8*d* shows that the fish do not exhibit chaotic leadership switching.

### 3.5. Quadratic drag

Many design choices were made when developing the model. However, the main alternative assumption that requires further investigation is the effect of having velocity decrease in proportion to *v*^2^, as this is the natural choice from a hydrodynamical perspective [19], in particular for large fish. We now examine the model where both terms in the equation for d*v*/d*t* are squared. This provides the same solution for the nullcline (d*v*/d*t* = 0), and we have the following set of equations:

3.8 $$\frac{db}{dt}=b-\frac{b^{3}}{3}+c-v$$

and

3.9 $$\frac{dv}{dt}=a^{2}{\left(\frac{\arctan(z_{1}(b-b_{0}))}{\pi }+\frac{1}{2}\right)}^{2}\;-\;{\left(kv\right)}^{2}.$$

**Figure 9.**
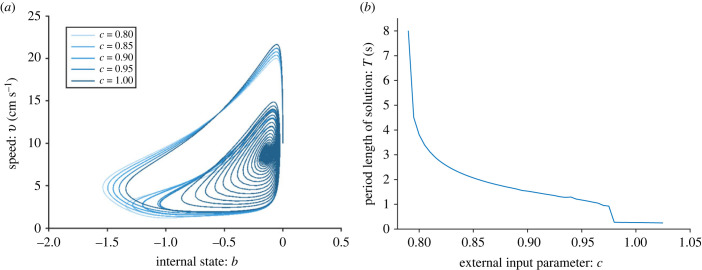
One-fish model with *k* = 0.8, *a* = 15 cm s^−2^, *z*_1_ = 50, *b*_0_ = 0 and quadratic drag where both terms in the equation for d*v*/d*t* are squared. Panel (*a*) shows the phase plane for five different values of *c*, as shown in the legend. For c≲0.79, the dynamical system has a stable equilibrium, resulting in constant speed. For c≳0.79, there is a stable limit cycle. As *c* increases the amplitude of the speed peaks increases, until c≳0.98 where the oscillation decays to a small circular limit cycle. Panel (*b*) shows the period length as a function of *c*. Increasing *c* leads to decreasing period length and thus increasing frequency.

In order to obtain a similar range of speeds, the speed decay was doubled to *k* = 0.8. While the nullclines are now identical, the phase-space trajectories are still of a different shape to the linear case, as can be seen in figure 9*a*. There is a threshold for bursting for a single fish at *c* ≈ 0.79, after which the bursting period decreases monotonically, until at *c* ≈ 0.98 where a canard explosion takes place [67].

**Figure 10.**
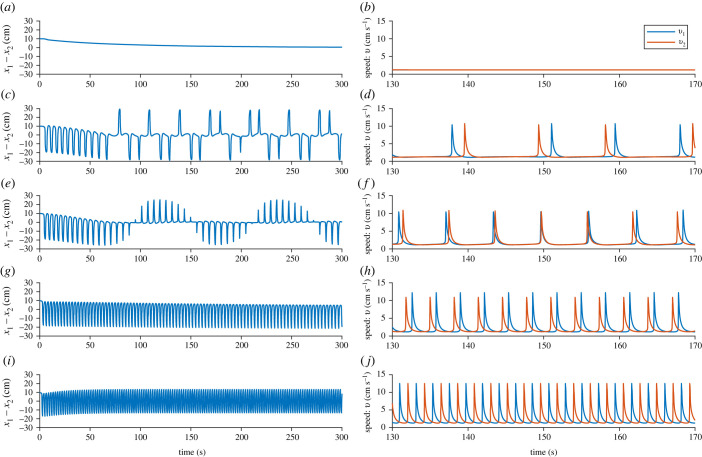
Two-fish quadratic-drag model with *a* = 15, *c* = 0.7885, *z*_2_ = 0.15, *x*_0_ = 15, *b*_0_ = 0, *z*_1_ = 50 and *k* = 0.8. In (*a*) and (*b*) where *d* = 0.0025, after one speed burst each, both fish move with constant speed and stay exactly parallel; in (*c*) and (*d*) where *d* = 0.0125, there is aperiodic leadership switching; in (*e*) and (*f*) where *d* = 0.025, there is periodic leadership switching; in (*g*) and (*h*) where *d* = 0.1, there is always one fish that is the leader; and in (*i*) and (*j*) where *d* = 0.2, there is no leader.

For interacting pairs, the same sequence of behaviours as the linear drag case is observed as *c* increases, as shown in figure 10. However, the speed bursts are clearly sharper than for the linear case.

Another design choice we made, which is worth mentioning here, is the choice of arctan function for *g*(*b*). This appears to be a necessary choice to get the dynamics we report here. When we tried different sigmoid functions for *g*(*b*), e.g. a logistic function and the Hill function, the two-fish model did not display the rich dynamics described in §3.

## 4. Discussion

The primary contribution of this paper is to show that burst-and-glide behaviour in fish can be reproduced by a burst-and-recover model of neurons. The fish velocity is viewed as being analogous to the recovery variable in the FitzHugh–Nagumo model for neural dynamics, and the internal state is analogous to the membrane potential variable. The model reproduces, for different parameter values, many aspects of the intermittent locomotion found in many species of fish [14–18]. In particular, for low coupling the fish move with a constant speed. For slightly higher values of the coupling parameter, *d*, the fish move by burst-and-glide swimming and switch leadership irregularly. When increasing the strength of the coupling further, the fish switch leaders at regular intervals. For even stronger coupling, there is always one leader, with the leader depending on the initial distance between the fish, similar to that seen in pairs of eastern mosquitofish [28]. These results can be replicated with quadratic drag, but the burst periods are much steeper, which makes the model less biologically realistic for guppies, but might capture the interactions of larger fish species.

In three of the four distinct forms of leader–follower dynamics, the follower fish bursts approximately 1 s after the leader (with the exact timing depending on parameter values), and has a higher maximum speed than the leader. However, the distance swum within one burst-and-glide period by the follower is always (slightly) shorter or the same distance as the distance swum by the leader within one burst-and-glide period. These qualitative model predictions would be relatively easy to test and may provide support for our model. For even higher values of the coupling parameter, the fish alternate positions and have the same maximum speed and distance travelled within one burst-and-glide period. When one fish reaches its minimum speed, the other reaches its maximum speed.

Another testable prediction arising for our model lies in the shape of the response function *f*. Previous experimental studies suggest that the response in speed is symmetric in the position of the other fish: the fish respond with a high speed both when the other fish is in front and behind [27]. As seen in figure 4*b*, our model reproduces the same results but without that assumption. This emphasizes the contributions of a mathematical model like ours: without a model, the natural assumption from experimental data are that the bursting response is as strong when the other fish is behind as when in front [47,68,69]. However, we find that a highly asymmetric response function *f*, for which the fish responds less strongly to a conspecific behind it than in front, also gives rise to the sort of dynamics observed in experiments, when coupled with the delay induced by the ‘recovery’ of the neuronal firing. This finding is reminiscent of the result of Perna *et al.*, who showed that (in models) two very different response functions can give rise to the same behaviour [70]. In our case, the recovery process gives the impression that the fish in front is responding to the fish behind, while in fact it has simply completed a burst-and-glide cycle, and is ready to burst again. The hypothesis arising from our model is also supported empirically by turning decisions measured in fish [32,71]. It could be tested more directly by, for example, studying isolated fish responses to a robot fish [72].

Quantitative agreement between our model and experimental data can be increased in at least three ways. Firstly, the data used was collected in a two-dimensional arena. Although we filtered the data to include times of near-parallel swimming, the angle between the fish headings is expected to have a distorting effect on the phase plot generated. An experiment conducted in a narrow channel or torus would be useful for comparison. Secondly, the motion in the experimental data was noisier than the model. Stochastic terms may help in this case to model noisy perception and/or action. In this case, a simple independent and identically distributed (i.i.d). error term added to the argument of *f*() and *g*(), respectively, may suffice. Thirdly, the comparison would also benefit from a rigorous parameter fitting procedure, as the non-bifurcation parameters have some influence on the shape of the phase-plane trajectories in a given behavioural mode, in addition to scaling of time and distance.

Existing models of leadership switching in fish include a random component to allow the switching to happen. For example, Markov chain models are often used to match experimental data of leadership switching [29,73,74]. Our model demonstrates that leadership switching can occur in a purely deterministic interaction. The irregular leadership switching in our model apparently results from a form of deterministic chaos. The Lorenz map (figure 6*a*) shows a clear deterministic form, while the autocorrelation indicates rapid decay in the correlation of movements. Evidence for chaos in animal behaviour has previously been presented in, for example, activity of single *Leptothorax allardycei* ants [75] and colonies of foraging ants [76].

Chaotic dynamics is a crucial part of neural mechanisms [39–44] and there are existing models connecting sensory systems to neuronal dynamics [55]. Our model appears to be the first in which chaos comes explicitly from a social interaction in a perception–action loop, rather than internally from the neurons. Chaos arises from the coupling between the intake of visual stimuli and the internal dynamics of the fish response. There are increasing experimental studies linking neuronal activities in the brain to locomotion in fish [56,57,77]. Recent advances in, for example, tilt in place microscopy [78], could allow measurement of brain activity in the presence or the absence of other fish or of stimulus that mimics interactions with other fish [79,80]. In the context of our theoretical work, it would be interesting to look for a potentially chaotic attractor in brain activity that is then influenced by interactions between fish.

While leadership switching is reasonably widespread in Nature, in our model it occurs when the coupling *c* is close to the bifurcation point of the one-fish model. Indeed, there is a very narrow range of parameters where the fish movements are chaotic. If leadership switching is ubiquitous in Nature, then one explanation for this might be an evolution of the neural parameter toward the Hopf bifurcation point at which burst-and-glide behaviour emerges. Similar mechanisms have been suggested in mathematical models describing foraging of ants and decision-making in slime mould [81,82]. The argument in this case is that being near to a bifurcation point offers flexibility in foraging. Similarly, it has been argued that biological chaos is generally favoured by selection [83]. In our case, such an argument would suggest that evolutionary pressure takes fish responses towards the chaotic switching regime, where the fish regularly switch leadership.

While burst-and-glide swimming is pivotal in single fish and pairs of fish, intermittent locomotion becomes less evident in schools of fish. Instead, leadership becomes less apparent and fish move more smoothly and are synchronized when schooling. A future direction of our work would thus be to understand how this transition from turn-taking to coordinated movement occurs as the number of fish in a shoal increases.

## Data Availability

Data and code are available from the GitHub repository: https://github.com/linneagyllingberg/neuronal-fish-dynamics.
